# The association between improved quality diabetes indicators, health outcomes and costs: towards constructing a “business case” for quality of diabetes care - a time series study

**DOI:** 10.1186/1472-6823-14-92

**Published:** 2014-12-01

**Authors:** Rachel Wilf-Miron, Arkadi Bolotin, Nesia Gordon, Avi Porath, Ronit Peled

**Affiliations:** The Gertner Institute for Epidemiology and Health Policy Research, Ramat, Gan, Israel; The School of Public Health, Sackler Faculty of Medicine, Tel Aviv University, Tel Aviv, Israel; Department of Public Health, Faculty of Health Sciences, Ben Gurion University of the Negev, Beer Sheva, Israel; Central Administration, Maccabi Healthcare Services, Tel Aviv, Israel; Maccabi Institute for Health Research, Tel Aviv, Israel; Epidemiology Department, Ben Gurion University of the Nege, Beer Sheva, Israel; Department of Health Systems Management, Faculty of Health Sciences, Ben Gurion University of the Negev, Beer Sheva, Israel

## Abstract

**Background:**

In primary health care systems where member’s turnover is relatively low, the question, whether investment in quality of care improvement can make a business case, or is cost effective, has not been fully answered.

The objectives of this study were: (1) to investigate the relationship between improvement in selected measures of diabetes (type 2) care and patients’ health outcomes; and (2) to estimate the association between improvement in performance and direct medical costs.

**Methods:**

A time series study with three quality indicators – Hemoglobin A1c (HbA1c) testing, HbA1C and LDL- cholesterol (LDL-C) control - which were analyzed in patients with diabetes, insured by a large health fund. Health outcomes measures used: hospitalization days, Emergency Department (ED) visits and mortality. Poisson, GEE and Cox regression models were employed. Covariates: age, gender and socio-economic rank.

**Results:**

96,553 adult (age >18) patients with diabetes were analyzed. The performance of the study indicators, significantly and steadily improved during the study period (2003–2009). Poor HbA1C (>9%) and inappropriate LDL-C control (>100 mg/dl) were significantly associated with number of hospitalization days. ED visits did not achieve statistical significance. Improvement in HbA1C control was associated with an annual average of 2% reduction in hospitalization days, leading to substantial reduction in tertiary costs. The Hazard ratio for mortality, associated with poor HbA1C and LDL-C, control was 1.78 and 1.17, respectively.

**Conclusion:**

Our study demonstrates the effect of continuous improvement in quality care indicators, on health outcomes and resource utilization, among patients with diabetes. These findings support the business case for quality, especially in healthcare systems with relatively low enrollee turnover, where providers, in the long term, could “harvest” their investments in improving quality.

## Background

During the last two decades, the quality of care has drawn increasing attention of health care systems. Among the cornerstones shaping this interest, were the inception of the Healthcare Effectiveness Data and Information Set (HEDIS) tool, which became available in 1993, with annual reporting of community-based performance measures of 90% of US health plans [1]; a series of articles on the quality of health care, published in the New England Journal of Medicine in 1996, illustrated how changes in measurement and quality improvement may affect physicians and patients over the coming decade [2]; and the 2001 Institute of Medicine (IOM) report, claiming that the US healthcare system needs to be re-designed in order to cross the “quality chasm” [3].

The term “Business Case” has been used to convince decision makers to invest in short-term actions which could lead to substantial benefits in the long term period of time. However, the lack of return on investments within a reasonable time period, has been an obstacle to making a business case for improving health care quality [4].

System-wide reengineering of care in the American Veterans Affairs (VA) healthcare system has resulted in significant improvements in diabetes care indicators [5, 6]. In 2004, the British Government introduced a pay-for-performance and quality monitoring scheme with 136 indicators for family practices. The indicators covered management of chronic disease, practice organization, and patients’ experiences with respect to care [7]. This scheme was associated with improvements in recorded quality of diabetes care in the first year; modest improvements in subsequent years; and with variation in care between population groups which diminished under the incentives, but remained substantial in some cases [8].

The Israeli National Quality Measurement Program (IOM) [9] has demonstrated substantial improvements in most diabetes care indicators during the past years, compared with those of the American HEDIS [10, 11].

Numerous studies have shown the effect of organizational or local efforts on performance indicators; however, fewer studies have looked at the effect of improvement in performance indicators on improvement in either patients’ health outcomes [12, 13] or medical costs. A recent publication by the IOM claims that the US healthcare system had become too complex and costly to maintain business as usual. The report suggests that transformational changes are required to shape a health care system which delivers the best care at the lowest cost [14].

Maccabi Healthcare Services (MHS) is the second largest Israeli health plan, providing community-based health services to two million members throughout the country. Service is delivered and managed by five regions through 150 branches. MHS, like other Israeli health plans, enjoys fully computerized health records. As a provider of community-based primary and secondary health care, MHS contracts with local hospitals to purchase tertiary care. The cost of hospitalization services is the single largest component in the MHS’ budget (41.2% of its expenses in 2009).

In 2004, MHS implemented a “Performance Measurement System” (PMS), which helped focus the organization’s attention on selected clinical domains, including diabetes care. To encourage care improvement, annual targets were set for all units, with modest rewards given to units achieving their targets. Additionally, MHS has developed quality infrastructures at the central and regional levels to encourage adoption of quality concepts and tools and allow for organizational learning from success and failure.

Diabetes, which is a major chronic disease, is one of the clinical domains which has attracted intense MHS effort to improve the care provided to its members [15, 16]. As evidence, 5 of the 24 indicators monitored by MHS’ PMS are related to diabetes care.

**The objectives** of this study were: (1) to investigate the relationship between improvement in selected measures of diabetes (type 2) care and patients’ health outcomes; and (2) to estimate the effect of improved performance on direct medical costs.

## Methods

### Study design

Time series study with repeated measurements.

### Setting

The study was conducted by MHS, using retrospective data for the period 2003–2009.

### Focus of analysis

Two intermediate outcome (HbA1C and LDL-Cholesterol control among patients with type 2 diabetes) and one process (HbA1C testing) measures were analyzed. Indicator selection was based on availability of complete longitudinal data and “stable” definitions (i.e., measure definition did not change during the period covered by the data). Health outcomes were represented by number of hospitalization days, Emergency Department (ED) visits and mortality.

### Study population

All MHS members aged 18 and above listed in the MHS Diabetes Registry (DR), between 2003 and 2009 [17]. Mean time of follow up was 8.5 (±1.7) years.

### Data source

HbA1C and LDL cholesterol (LDL-C) test results and documentation of HbA1C testing were retrieved from the MHS PMS. Number of hospitalization days and ED visits (per patient, per year), time of death (date), age, gender and socio-economic rank (SER) were retrieved from the MHS central databank.

### Quality indicator definitions

HbA1C testing: Documented at least once during the measurement year; poor Glycemic control: HbA1C > 9% in the last recorded test for each year; LDL-C control: LDL-C < 100 mg/dl in the last recorded test for each year. All indicators were based on the American HEDIS definitions [1].

### Other definitions

#### SER

The socio-economic rank of the member’s residential neighborhood for the period studied, measured on a 10-point scale, were retrieved from the census data kept by the Israel Central Bureau of Statistics [18].

### Statistical analysis

Linear regression models were employed in order to evaluate the change in performance of selected indicators between the years 2003 and 2009.

#### Repeated measurement data set structure

The data set comprised for each DR registered patient all the information under investigation, for each year (2003–2009 = 7 lines 2009–2012 death information = additional 3 lines).

*Poisson regression models* were employed in order to account for the prevalence of zero counts in the data. The zero-inflated (or zero-altered) Poisson model allows over dispersion, through the splitting process, that models the outcomes as zero or nonzero.

Additionally, *Generalized Estimating Equations* (GEEs) were employed. GEEs fit generalized linear models of the dependent variable *y*_*it*_ (in our case: hospital days) with covariates *x*_*it*_.


for *i* = 1, …, *m* and *t* = 1, …, *n*_*i*_, where there are *n*_*i*_ observations for each group identifier *i* (in our case, the patient’s ID). *g*( ) is called the link function, and *F* is the distributional family. Substituting various definitions for *g*( ) and *F* result in a wide array of models. In our case, *y*_*it*_ is assumed to be distributed negative binomial and *g*( ) is log, therefore:


yielding log-linear regression related models, depending on what is assumed for the within-group (within the particular patient’s records, in our case) correlation structure.

Let ***R*** be the working correlation matrix for modeling the within-group correlation, a square *max*{*n*_*i*_} × *max*{*n*_*i*_} matrix. Let ***R***_*t*,*s*_ denote the *t, s* element. We assumed that the working correlation matrix is *independent*, therefore:


Using GEEs with the independent correlation matrix corresponds to exploiting cluster option in General Linear Models (in our case, cluster is a set of the particular patient’s records).

*The global model* takes the “time” as a reference point, while the *dynamic model* takes the variations in “time” along the study period. Global models were employed for hospitalization days and death. Dynamic models were employed for hospitalization days.

To evaluate association between the study year and HbA1C levels, the interaction variable “HbA1C_year” was computed by multiplying HbA1c indicator (i.e., the binary, 0-or-1, variable that equals 1 if HbA1c is greater than the fixed at the particular year level of HbA1c) by the sequel year (starting with 0 as the year of entering into the study and going up with follow-up years).

To estimate the effect on death, a Cox Regression model was employed. This model fits the hazard, assuming an exponential function of the summation of the regression coefficients b_1_,…,b_k_. The Cox model provides estimates of b_1_,…,b_k_ but provides no direct estimate of the baseline hazard. For this task, data on death events were retrieved from MHS’ central warehouse, and the years 2010–2012 were added.

To estimate average proportion of annual reduction in hospitalization days (saving days), the coefficient of dynamic model minus the coefficient of global model was calculated. To calculate cost savings, the number of hospitalization days saved was multiplied by cost per hospitalization day as noted on the Israeli Ministry of Health price list [19]. This price list is based on an average of direct cost between the internal and other general wards and corrected to the year of data analysis.

All models were adjusted for age, gender and SER considered as potential confounders. To analyze the data, STATA ver. 12 and SPSS ver.20 software were used. Alpha level for statistical significance was 0.05.

## Results

The analysis included data collected on 96,553 registered adult patients with diabetes. Mean age was 64.3 (±13.9), 43.5% were females and 64.7% belonged to the higher SERs (ranks 6–10) (Table 1).

**Table 1:** **Study population characteristics**

Variable	N = 96,553
Mean Age (years)	64.3 (±13.9)
Females (%)	43.5
SER* (%)	
1-5	29.4
6-10	64.7
No available data	5.9
Mean time of follow up	8.5 (±1.7) years

*SER: Socio-Economic Rank.

During 2003–2009, rates of HbA1C testing increased from 43.8% to 69.6%; rates of HbA1C poor control decreased from 13% to 9.2%, and rates of LDL-C control increased from 29.4% to 57.7% - all statistically significant (Table 2).

**Table 2:** **Performance rates (%) of selected indicators, 2003-2009**

Indicator	2003	2004	2005	2006	2007	2008	2009	P value*
HbA1C > 9%	NM^†^	13.0	11.1	11.7	11.1	9.3	9.2	0.010
LDL Control <100 mg/dl	29.4	30.9	37.7	47.2	50.5	56.7	57.7	<0.001
HbA1C testing	43.8	49.5	54.5	59.0	62.3	64.8	69.6	<0.001

*linear regression models; ^†^Not measured.

The Poisson model for hospitalization days (as the depended variable), revealed a significant association (P < 0.0001) with poor HbA1C and LDL control (Table 3).

**Table 3:** **Results of Poisson regression models for hospitalization days (dependent variable), adjusted for age, gender and SER**

Variable	Coefficient	S.E.	95% CI*	P value
**Hospitalization days**				
HbA1C >9%	-0.059	0.015	-0.089- -0.028	<0.001
LDL >100 mg/dl	0.034	0.002	0.029-0.040	<0.001

*95% confidence interval; S.E = Standard Error.

Both the global and dynamic GEE models revealed a positive and significant association between poor HbA1C control (>9%), lack of LDL-C control (>100 mg/dl) and number of hospitalization days (Table 4).

**Table 4:** **Results of three GEE models for hospitalization days and mortality (dependent variables) adjusted for age, gender and SER**

Variable	Coefficient	S.E.	95% CI	P value
***Global Model*** **Hospitalization days**				
HbA1C >9%	5.35	0.63	4.1-6.6	<0.001
HbA1C > 9%*Year	-0.50	0.07	-0.64- -0.35	<0.001
LDL >100 mg/dl	0.13	0.05	0.02-0.24	0.020
**Mortality**				
HbA1C >9%	0.55	0.04	0.45-0.64	<0.001
LDL >100 mg/dl	0.20	0.03	0.14-0.27	<0.001
***Dynamic Model*** **Hospitalization days**				
HbA1C >9%	5.75	0.67	4.2-6.8	<0.001
HbA1C >9% *Year	-0.52	0.07	-0.67- -0.36	<0.001
LDL >100 mg/dl	0.13	0.05	0.018-0.24	0.022

*95% confidence interval.

The interaction with time (year) suggested that the proportion of patients with poor HbA1C and LDL control consistently and significantly decreased each year (Table 4). HbA1C testing did not reach statistical significance and was thus removed from further models (P = 0.785).

The models for ED visits as a dependent variable were too weak (LR Chi^2^ = 73.4) to demonstrate any association with the independent variables.

Death as a dependent variable was positively and significantly associated with the following indicators: poor HbA1C control and inappropriate LDL-C control (>100 mg/dl). The hazard ratio for time of death (as a dependent variable) for HbA1C > 9% and LDL-C > 100 mg/dl indicators was 1.78 and 1.17 (P < 0.001), respectively (Table 5).

**Table 5:** **Cox regression model for time to death (dependent variable), adjusted for age, gender and SER (2003–2012)**

Variable	Hazard ratio	S.E	95% CI ^†^	P value
HbA1C > 9%	1.78	0.08	1.63-1.95	<0.001
LDL > 100 mg/dl	1.17	0.039	1.09-1.25	<0.001

Notes: ^†^95% Confidence Interval.

Calculation of the average annual drop in the proportion of patients with HbA1C > 9% (0.52-0.50) revealed an average annual saving of 2% in hospitalization days, and a meaningful saving in hospitalization costs (Table 6).

**Table 6:** **Saving of hospitalization days due to improvement in HbA1C > 9% indicator 2005–2012**

Year	Hospital days	Saving	Saving in NIS*
2005*	94,447	1,927	3,944,000
2006	137,325	2,802	5,604,000
2007	176,131	3,594	7,188,000
2008	200,487	4,091	8,182,000
2009	195,606	3,991	7,982,000
2010	219,813	4,485	8,970,000
2011	205,563	4,195	8,390,000
2012	110,851	2,262	4,524,000

1NIS (Israeli Shekel) = 3.6 US$.

Note: *Since measurement and systemic quality improvement efforts were initiated in 2004, we calculated the decrease in hospitalizations as result in improved diabetes control from 2005. The effect of improved control in 2009 is expected to have an effect on hospitalization at least until 2012.

## Discussion

### Key findings

Our study has demonstrated that over a 6-year-period, improvement in glycemic and cholesterol control was associated with significant decreases in hospitalization days, mortality and direct medical costs. ED visits failed to demonstrate a similar association.

#### Results in context

These results may be explained by the fact that improved glycemic and cholesterol control were important elements in the organization’s efforts to improve quality of care, through multidisciplinary health promotion programs created within the framework of a performance monitoring system that implemented the organization’s “call for action” [16].

In the early 1990s, the Institute of Medicine (IOM) [20, 21] defined quality as the “degree to which health services for individuals and populations increase the likelihood of desired health outcomes and are consistent with current professional knowledge”. Quality of care can then be evaluated on the basis of structure, process, or outcome [22]. When used appropriately, both process and outcome measures can provide valid information about quality objectives [23].

The decision to choose hospitalizations, ED visits and mortality as health outcomes (the dependent variables) was based on data availability. Data on diabetes complications and long-term trends in diabetic patient satisfaction remain unavailable. It has been long claimed that the use of outcome measures is insufficient for quality of care assessment since most differences between patients receiving the same treatment result from factors associated with patients’ characteristics – which are outside the control of health care providers [23]. Following our experience [24], it is believed that beyond the differences in personal characteristics, differences in care provided to MHS members tend to be marginal. However, differences in personal characteristics (gender, age and socio-economic rank) were adjusted in all the statistical models. It is suggested that further research will be conducted with a control for patient characteristics such as self-efficacy, self-capability to manage the disease, compliance with medical regiment, and so forth.

Data obtained from the Israeli National Program for Quality Indicators (NPQI) in Community Healthcare [9] indicated a 0.25% annual increase in diabetes prevalence from 2004 to 2010. National census data indicated that diabetes-related mortality rates, adjusted for ethnicity and gender, have decreased by 24% from 2004 to 2010 [25], most probably due to better control of intermediate outcome measures and better care for diabetes complications. Although data on diabetes-related hospitalizations are not available, crude national figures indicate a steady increase in hospitalization days between 2000 and 2010 [26].

It is worth mentioning that the NPQI, instituted in 2004, contributed to each of the four Israeli health plans, including MHS, for development of quality improving infrastructures, which resulted in improved performance indicators in most measured domains. It is, therefore, suggested that the observed continuous improvement in the selected measures presented here is not exclusively the result of “natural improvement”.

MHS’ performance monitoring system was a necessary but insufficient element for the explanation of long-term care improvement. Regional “Quality Teams”, comprised of physicians, nurses and other health professionals in managerial positions, were set up and trained to guide analysis of quality gaps and implementation of effective interventions. Resources were allocated to intervene in units which had exhibited wide gaps between actual performance and desired targets. Considerable effort has been invested in empowering patients throughout programs to increase treatment adherence [27], among other steps taken [16].

Our study also demonstrated association between significant reduction in mortality and improved glycemic and cholesterol control. Data from the United Kingdom has shown that the mortality risk among patients with Type 2 Diabetes is 1.6 times higher than that of the general population [28]. Landman and colleagues have reported that patients with diabetes evidencing poor glycemic control (HbA1C > 9%) exhibited a hazard ratio of 2.21 for total mortality, compared with a hazard ratio of 1.0 among the control group with normal glycemic control levels (HbA1C < 6.5%). This suggests that in order to increase life expectancy, interventions should focus mainly on patients evidencing poor control [29]. The literature also shows that correction of dyslipidemia (such as control of LDL-C) in patients with diabetes promotes reduction of macro-vascular disease, which contributes to cardio-vascular complications and shortened life span [30].

The models for ED visits (as a dependent variable) calculated in this study were too weak to produce significant statistical results. HbA1C performance (as an independent variable) was not found to be associated with either hospitalization days or death. It seems logical that this process variable is insufficiently powerful to explain these two outcomes. Additionally, HbA1C is measured as performed at least once a year; the findings indicate that testing only once a year is insufficient for disease control and achievement of desired outcomes.

Providing appropriate care for patients with diabetes, especially those exhibiting complications resulting from poor disease control, demands considerable resources. Improving the quality of care to patients with diabetes and achieving better health outcomes is also costly [15, 31–33]. Moreover, health care systems around the world are facing pressure to constrain costs, given the rise in medical sophisticated technologies and the aging of the population, among other reasons. Those trends prompt health care organizations’ decision makers to expect a “business case” for quality improvement, meaning that these investments would have a “return on investment” (ROI) within a reasonable period of time. The annual average result of 2% reduction in hospitalization days through the reduction in poor glycemic control is a preliminary pivotal evidence for such a business case. The results comply with the results of several other studies [34–38], whose authors conclude that sustained reduction in HbA1C levels among patients with diabetes is associated with significant cost savings within 1–2 years of level reduction. MHS, as all other Israeli HMOs, is characterized by a very low patient turn over, meaning that only a small portion of the population leaves its HMO during its life time. This fact urges the Israeli HMOs to invest in quality improvement, knowing that they can return their investments in the long term.

Blumenthal and colleague [4] argue that a leading obstacle to achieving quality in health care is the absence of a “*business case”* for quality. Healthcare system infrastructure is frequently accused of being inadequate to support such thinking. Furthermore, one of the root causes mentioned is the primitive quality measurement stage of science; if most healthcare providers are unable to estimate the total cost of investing in quality, how can one expect them to calculate the savings produced by their investment in interventions? Also, interventions to improve diabetes care produce return on investment only in the medium- to long-term (delayed savings). Therefore, healthcare organizations with a high turnover may not be able to achieve this return [39]. In times of austerity, the majority of budget cuts take place in the healthcare sector [40], which adds pressure on organizations to economically “justify” quality improvement investments.

#### Study’s strengths and limitations

The strength of this study lies in the fact that data were analyzed for a very large study population (N = 96,553) comprising all adult members of a major health plan, registered in the Diabetes Register, which increases the statistical validity of the findings. In addition, a robust statistical techniques were employed to support the hypothesized results.

Yet, the study has considerable limitations, which should be overcome in future studies: (1) The effect of only three diabetes performance measures was studied. Appropriate follow up with process measures such as eye and foot examinations or medical attention for nephropathy as well as intermediate blood pressure control were not included in the analysis because the measure’s definition changed during the study period or the measure was not documented throughout the study period; (2) our information system, although fully computerized, was not designed to collect accurate and full data on the costs of quality improvement interventions or medical expenditures directly related to diabetes care; hence, the “investment” side of the “business case” argument was not thoroughly looked at. The data cannot be appropriately used to substantiate returns on investments in quality. Furthermore, the cost of hospitalization is subject to local agreements and contracts between HMOs and hospitals, and is not publicly transparent. The estimated cost saving is, therefore, based on the price list published by Israel’s Ministry of Health, which may not fully reflect actual prices. 3). This study investigated the entire MHS’ population; thus, a control group was not available, as all MHS members were under the quality improvement scheme. A further investigation with a case control design is recommended.

## Conclusion

In conclusion, our study indicates the effect of continuous improvement in performance indicators on health outcomes and resource utilization among patients with diabetes. In health care systems with relatively low member turnover and beneficiaries who do not leave their HMO for a long period of time, this finding represents an important milestone, linking quality and cost, helping to construct the business case approach to quality; these conclusions may convince managers to invest scarce resources in care improvement.

### Ethics

This study was a result of the routine analysis of data and did not required an ethical approval.
